# Safety and Efficacy of Dihydroartemisinin-Piperaquine in Falciparum Malaria: A Prospective Multi-Centre Individual Patient Data Analysis

**DOI:** 10.1371/journal.pone.0006358

**Published:** 2009-07-29

**Authors:** Julien Zwang, Elizabeth A. Ashley, Corine Karema, Umberto D'Alessandro, Frank Smithuis, Grant Dorsey, Bart Janssens, Mayfong Mayxay, Paul Newton, Pratap Singhasivanon, Kasia Stepniewska, Nicholas J. White, François Nosten

**Affiliations:** 1 Shoklo Malaria Research Unit, Mae Sot, Thailand; 2 Faculty of Tropical Medicine, Mahidol University, Bangkok, Thailand; 3 Centre for Tropical Medicine, Nuffield Department of Clinical Medicine, University of Oxford, CCVTM, Oxford, United Kingdom; 4 Médecins Sans Frontières - Holland, Yangon, Myanmar; 5 Wellcome Trust – Mahosot Hospital – Oxford Tropical Medicine Research Collaboration, Mahosot Hospital, Vientiane, Laos; 6 National Malaria Control Programme, Kigali, Rwanda; 7 Institute of Tropical Medicine, Antwerp, Belgium; 8 Médecins Sans Frontières- Belgium, Phnom Penh, Cambodia; 9 Department of Medicine, University of California San Francisco, San Francisco, California, United States of America; 10 Department of Postgraduate Studies and Research, University of Health Sciences, Vientiane, Laos; Sabin Vaccine Institute, United States of America

## Abstract

**Background:**

The fixed dose antimalarial combination of dihydroartemisinin-piperaquine (DP) is a promising new artemisinin-based combination therapy (ACT). We present an individual patient data analysis of efficacy and tolerability in acute uncomplicated falciparum malaria, from seven published randomized clinical trials conducted in Africa and South East Asia using a predefined in-vivo protocol. Comparator drugs were mefloquine-artesunate (MAS3) in Thailand, Myanmar, Laos and Cambodia; artemether-lumefantrine in Uganda; and amodiaquine+sulfadoxine-pyrimethamine and artesunate+amodiaquine in Rwanda.

**Methods and Findings:**

In total 3,547 patients were enrolled: 1,814 patients (32% children under five years) received DP and 1,733 received a comparator antimalarial at 12 different sites and were followed for 28–63 days. There was no significant heterogeneity between trials. DP was well tolerated with 1.7% early vomiting. There were less adverse events with DP in children and adults compared to MAS3 except for diarrhea; ORs (95%CI) 2.74 (2.13 to 3.51) and 3.11 (2.31 to 4.18), respectively. DP treatment resulted in a rapid clearance of fever and parasitaemia. The PCR genotype corrected efficacy at Day 28 of DP assessed by survival analysis was 98.7% (95%CI 97.6–99.8). DP was superior to the comparator drugs in protecting against both P.falciparum recurrence and recrudescence (P = 0.001, weighted by site). There was no difference between DP and MAS3 in treating P. vivax co-infections and in suppressing the first relapse (median interval to P. vivax recurrence: 6 weeks). Children under 5 y were at higher risk of recurrence for both infections. The proportion of patients developing gametocytaemia (P = 0.002, weighted by site) and the subsequent gametocyte carriage rates were higher with DP (11/1000 person gametocyte week, PGW) than MAS3 (6/1000 PGW, P = 0.001, weighted by site).

**Conclusions:**

DP proved a safe, well tolerated, and highly effective treatment of P.falciparum malaria in Asia and Africa, but the effect on gametocyte carriage was inferior to that of MAS3.

## Introduction

Over 80 countries worldwide have now implemented WHO recommendations to use artemisinin-based combination therapy (ACT) as first-line treatment of *Plasmodium falciparum* malaria [1], [2]. Dihydroartemisinin-piperaquine (DP) is a fixed dose co-formulated ACT used increasingly in South East Asia, although it is not yet registered by most national drug authorities. Most experience of the use of DP comes from Vietnam, where it is the recommended first-line treatment. The bisquinoline compound piperaquine as a monotherapy was used extensively in China where it replaced chloroquine as the first-line treatment of falciparum and vivax malaria. Between 1976 and 1994 over 300 tons of piperaquine were used in China in antimalarial prophylaxis and treatment. The first combination of DHA and piperaquine (China-Vietnam 8, CV8), also included primaquine and trimethoprim and was first evaluated in Vietnam in 1990 [3]. CV8 was effective, and became part of national treatment policy but, because of primaquine toxicity concerns and uncertainty whether trimethoprim contributed to treatment efficacy, these two component drugs were eventually removed. The new two drugs combination became first line treatment in Vietnam in 2007.

Piperaquine has a terminal half-life of several weeks [4]. It is highly active against chloroquine-resistant *Plasmodium falciparum*, and *vivax* [5]. Dihydroartemisinin (DHA) is the active metabolite of artesunate and artemether. Recently, several clinical trials have been carried out to study the safety and efficacy of DP for the treatment of *P.falciparum* malaria. The randomized trials included in this individual patient data analysis [6]–[12] were conducted between October 2003 and June 2006 using a prospectively predefined protocol with a follow-up of at least 28 days and use of PCR parasite genotyping to distinguish new infections from recrudescences.

## Methods

The trials were conducted in North-western Thailand, Rakhine state, Myanmar, Southern Laos, and Western Cambodia, where mefloquine combined with a three day course of artesunate (MAS3) was the comparator drug, in Uganda where artemether-lumefantrine (AL) was the comparator, and in Rwanda, where artesunate+amodiaquine (AS+AQ) and a non-ACT group amodiaquine+sulfadoxine-pyrimethamine (AQ+SP) were the comparator antimalarial drugs. In Rwanda, following high levels of chloroquine (CQ) resistance, the combination AQ+SP was adopted as the first-line anti-malaria treatment in 2001. However, AQ+SP has always been considered an interim strategy and different artemisinin-based combination treatments (ACT) have been tested in the past few years as possible alternatives.

Patients presenting with acute uncomplicated falciparum malaria were recruited into the treatment studies provided they gave fully informed consent. Eligible patients were 12 to 59 months old patients weighing more than 10 kg in Rwanda, 6 months to 10 years old, weight >5 kg in Uganda, and all patients between 1 and 65 years in Myanmar, Laos, Cambodia, and Thailand. Only uncomplicated cases of *P.falciparum* monoinfections were included in Laos, Rwanda, and Uganda, while in Cambodia, Thailand and Myanmar patients with mixed (*P.falciparum* and *P. vivax*) infections were included. Studies excluded pregnant or breastfeeding women, patients with HIV-AIDS or severe malaria. Malaria on admission and reappearance were confirmed by microscopy examination of blood smears. All sites screened actively for adverse events until Day 28.

### Study design and treatment regimens

Trials were open label randomized comparative studies. Lengths of follow-up varied from 28 days in Rwanda, to 42 days in Laos, Myanmar, Uganda, and 63 days in Cambodia and Thailand. Patients received a total dose of approximately 7 mg/kg bw dihydroartemisinin and 56 mg/kg bw piperaquine divided into 3 daily doses, except in Cambodia and in the first trial in Thailand in which an earlier dose regimen was used where the same total dose was divided into 4 doses given at 0, 8, 24 and 48 h. One tablet of DP (Artekin®, Holleykin Pharmaceutical Co., and in Uganda: Duo-cotecxin®, HolleyPharm) contained 40 mg of dihydroartemisinin and 320 mg of piperaquine phosphate. Comparator drug doses were MAS3 3 days: artesunate 4 mg/kg/day, and mefloquine 8 mg/kg/day, or AQ+SP: AQ 10 mg/kg/day for 3 days and SP 25 mg/kg of sulfadoxine and 1.25 mg/kg of pyrimethamine the first day or AS+AQ 3 days: artesunate 4 mg/kg/day, and amodiaquine 10 mg/kg/day, and AL, 20 mg artemether/120 mg lumefantrine tablets according to weight as one (5–14 kg), two (15–24 kg), three (25–34 kg), or four (≥35 kg) tablets given twice daily for 3 days.

Drug administration was observed directly by study investigators, except for one arm in Myanmar where effectiveness was assessed [8]. The doses were crushed and mixed with water and given in a syringe or on a spoon for children unable to swallow tablets, and if vomiting occurred within one hour of dosing (defined as early vomiting), the medication was re-administered. Drugs were given with food in Cambodia, and Laos, and a glass of milk in Uganda. In Myanmar, Thailand, and Rwanda no food was given.

### Reappearance of falciparum malaria during the follow-up period

Polymerase chain reaction (PCR) parasite genotyping was performed on paired samples for parasite genotyping to distinguish between new infections and recrudescent cases. Allelic variation within MSP1, MSP2, and GLURP was used in Asia as described previously [13]. In Rwanda, DNA was purified [14] and two polymorphic markers MSP1 and MSP2 were analyzed [15]. In Uganda where transmission intensity is very high, selected regions of MSP1 and MSP2 and 4 microsatellite markers were amplified using PCR and characterized based on sequence and size polymorphisms identified by gel electrophoresis [16].

### Ethical Approval

The clinical trials from each country were approved by appropriate authorities. The Thai studies were approved by the Faculty of Tropical Medicine, Mahidol University Ethical Committee (Bangkok, Thailand). Approval for the Laos study was granted by the Ethical Committee of the Faculty of Medical Sciences, National University of Laos; both of these studies were also approved by the Oxford Tropical Research Ethics Committee (OXTREC), University of Oxford, UK. The protocol for the trial in Myanmar was approved by the Myanmar Department of Health and by the Médecins Sans Frontières (MSF) Ethical Review Board. In Rwanda, the study was reviewed and approved by the Ministry of Health of Rwanda and by the Ethical Committee of the Prince Leopold Institute of Tropical Medicine, Antwerp, Belgium. The Cambodian study received ethical clearance from the Cambodian National Ethical Review Committee (Ministry of Health, Cambodia), and the MSF Ethical Review Board. In Uganda, approval came from the Makerere University Research and Ethics Committee, the Uganda National Council of Science and Technology, and the University of California, San Francisco Committee for Human Research.

### Data pooling

The databases of randomized controlled trials were sent by the investigators. The following aspects of the quality of trial methodology were evaluated: generation of the allocation sequence, adequacy of concealment of the allocation of treatment, degree of blinding, and completeness of follow-up. Generation of the allocation sequence and allocation concealment was classified as adequate, inadequate, or unclear [17].

Blinding was classified as open, single or double. The proportion of patients lost to follow-up (regardless of failures) was computed and considered acceptable if <10% within 28 days. Other markers of quality assessed were whether a sample size was determined using power calculations and whether an intention-to-treat (ITT) analysis could be computed.

### Study endpoints and statistical analysis

The analysis was by modified intention-to-treat where patients who did not complete the study were censored on their last day of follow-up, but they were not regarded as a failure as in a “pure” ITT analysis.

The primary endpoint was the treatment efficacy by Day 28. Patients lost to follow-up (or missing a weekly visit) or with a new *P. falciparum* infection were censored for the primary outcome at the time they were last seen. All studies followed patients for at least 28 days and the primary endpoint was defined prospectively as the parasitological treatment failure (PCR confirmed: recrudescence, and PCR not corrected: recurrence). Treatment failure was considered as the sum of early and late treatment failures, as defined by the WHO [1] as one of the following: (i) danger signs, death, or severe malaria at Days 1, 2 or 3 with parasitaemia; (ii) parasite density at Day 2>Day 0; (iii) parasitemia at Day 3>25% than Day 0, and recurrent parasitaemia after Day 4. Patients could be given less than the full dose if they received rescue treatment or withdrew consent from follow-up. Adequate Clinical and Parasitological Response (ACPR) was defined as no parasitaemia until the end of the follow-up without previously meeting any of the criteria for failure.

The efficacy was measured using Kaplan-Meier survival analysis. We applied a statistical correction for cases where PCR genotyping gave an indeterminate result or was unavailable, computing adjusted quotients by determining the probability by site and at any time of a parasite reappearance being either a recrudescence or a new infection [18].

### Secondary outcomes were:

-  The risks of recrudescence (reappearance of the same genotype) in the DP groups compared to the comparator groups (stratified by study), using the different lengths of follow-up of each studies.
-  The risks of recurrence (defined as recrudescence as above and new infections) in the DP groups compared to the comparator groups (stratified by study), using the different length of follow-up of each studies.
-  The risk of new infection (excluding recrudescences) in DP groups compared to the comparator groups per study and overall result (stratified by study) at Day 28.
  The risks of treatment failure (i, ii, iii) were compared in multivariate analysis (Cox regression models) individually and overall by stratifying by site in an attempt to account for potential statistical heterogeneity.
-  The predictors of recrudescence or new infection or recurrence by Day 28 were assessed by Cox regression models. The covariates examined were: sex, age (continuous), food absorption with the drug, anaemia on admission (haematocrit <30%), parasite count on admission (log transformed), elevated temperature on admission (temperature measured by any method at ≥37.5°). As the age groups were different between Africa (under 5 years old) and Asia (children and adults), we conducted this part of analysis separately for the 2 continents.
-  Gametocyte carriage
  - The predictors of gametocyte prevalence on admission were measured using a logistic regression and controlling for site.
  - The time to clearance of gametocytes already present on admission with data censored at the time of gametocyte clearance was calculated based on the results of blood smears. Within each study, the same sampling schedules were used for all the patients, but between trials parasite counts were performed at different times so any analysis was stratified by site to account for these differences. For patients who cleared gametocytaemia, time of the first negative count (followed by further negative counts) was taken as time of clearance. Differentials in gametocytes clearance were calculated using Kaplan-Meier method using logrank test, stratified by site, and a Cox regression model stratified by site measured the risks of gametocytes carriage between treatment groups.
  - The predictors of gametocyte appearance during the follow-up in patients without gametocytaemia on admission were assessed by a Cox regression model stratified by site. The presence of gametocytaemia after starting treatment was analysed as a binary variable: the Mantel-Haenszel method and the homogeneity test stratified by site were used to estimate a combined odds ratio between treatments. One positive gametocyte count at any time after treatment during the follow-up period was enough to define gametocyte carriage, while a complete set of negative counts during follow-up was required to confirm no carriage.
  - Gametocyte carriage rates were measured in person-gametocyte-weeks, using binary variable calculated within 42 days of follow-up. Person-gametocyte-weeks (PGW expressed per 1000) were defined as the number of weeks in which blood slides were positive for gametocyte divided by the total number of weeks followed up in patients with gametocyte results [19]. Mantel-Haenszel rate ratios (RR) weighted by site were used to measure to the risks between treatment groups.
-  Haematological changes were measured using the paired t-test. Anaemia was defined as haematocrit <30%, and anaemia recovery during the follow-up by the time for the haematocrit to reach 30% or more.
-  Adverse events: defined as any sign, symptom, or disease that was not present on admission and was associated with the use of a medicinal product, whether or not it was considered as related to the medicinal product. A serious adverse event was defined as a sign or symptom that was fatal, life threatening or required admission to hospital. Adverse events were standardised and expressed as an incidence density, in person-days at risk within 28 days. The incidence rate ratio test was used to compare the incidence of adverse events. We assumed that young children (<5 years old) were unable to answer questions about dizziness, nausea, headache, confusion, numbness, hearing disturbance, tinnitus or visual disturbance.
-  The effects of DP on *P. vivax* recurrences in the Asian trials with longer follow-up (63 days) was calculated by censoring the data at the time of *P. vivax* appearance (binary variable). The predictors of *P.vivax* appearance were measured by using Cox regression, and the incidence density of *P. vivax* appearance was calculated in person-day.

Heterogeneity was assessed by the Cochran Q test, and I^2^ test. Chi-square, Mann-Whitney, Kruskall-Wallis tests were used as appropriate. Confidence intervals (CI) were measured at 95% by the binomial distribution, or the Wilcoxon procedure, or the Taylor series estimate as appropriate [20]. The statistical programme used was STATA v10 (STATA corp.).

## Results

### Characteristics of included studies

**Figure 1:**
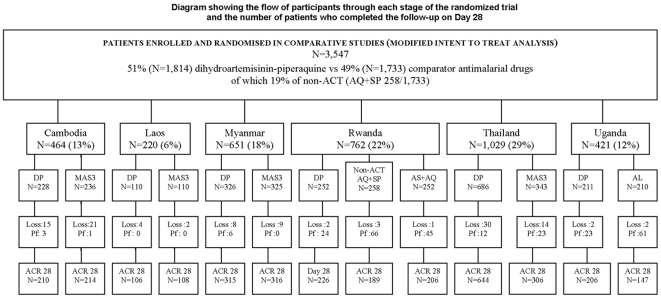
Note: DP; dihydroartemisinin-piperaquine, MAS3; mefloquine-artesunate, AQ+SP; amodiaquine+sulfadoxine-pyrimethamine, AL; artemether-lumefantrine, AS+AQ; artesunate+amodiaquine, Pf; *P. falciparum*, loss; loss to follow-up, ACR; adequate clinical response, ACT; artemisinin combination therapy.

A total of 3,547 patients were enrolled in six countries from 12 different sites between October 2003 and June 2006. Individually, the trials enrolled between 75 and 303 patients treated with DP (total = 1,814), 1,475 patients treated with other ACTs, and 258 in the non-ACT (AQ+SP) group (figure 1). The proportion of patients lost to follow-up was <10% in all the trials (4%, 110/3,547 at Day 28).

### Heterogeneity

In all the trials the methodological quality was high and the randomisation sequence was computer-generated. All trials were open label, and the basis for the sample size studied was provided in all studies. In all studies, the primary treatment outcome was the parasitological treatment failure. All trials reported data on haemoglobin levels during follow-up, and recorded gametocyte carriage at study enrolment and during follow-up. All studies assessed adverse events and 10 different adverse events were documented in all trials. Although there were differences in geographical location, transmission intensity (ranging from low and seasonal in Thailand to very high in the African trial settings), age, treatment and supervision, heterogeneity between trials was not significant (I^2^ test = 26%, P = 0.15, Cochran Q test for heterogeneity), and regarded as low [21].

### Baseline characteristics

The median age of the recruited patients was 13 years (range 1 to 65) (Table 1). Overall 32% of patients were under five years of age, 28% were 5–14, and 40% were adults. In Asia, age distributions were similar in Cambodia, and in Thailand. The patients were younger in Laos and Myanmar, while in Rwanda and Uganda only children were enrolled. There were no differences detected in admission characteristics between DP groups and comparator treatment groups, except for baseline gametocytaemia in Myanmar [8].

**Table 1:** Trials baseline characteristics, patients receiving dihydroartemisinin-piperaquine.

Country characteristics on admission			Cambodia		Laos		Myanmar		Rwanda		Uganda		Thailand		Total	
Total number of patients			228	13.4%	110	6.5%	327	19.2%	252	14.8%	211	12.4%	686	40.2%	1814	100%
Age, median in years (IQR; range)			21	(20; 2–65)	12	(17; 1–50)	7	(8; 1–42)	3	(2; 1–5)	2	(2; 1–9)	20	(20; 1–65)	12	(22; 1–65)
Age group	0–4	N, %	7	3.1%	19	17.4%	79	24.2%	248	98.4%	200	94.7%	31	4.5%	574	32%
	5–14	N, %	61	26.6%	49	45.0%	191	58.4%	4	1.6%	11	5.3%	190	27.7%	506	28%
	≥15	N, %	161	70.3%	41	37.6%	57	17.4%	0	0.0%			464	67.7%	723	40%
Male		N, %	160	69.9%	63	57.8%	165	50.5%	121	48.0%	97	46.0%	446	65.1%	1052	58%
Haematocrit (%)		Mean (SD)	35.5	(6.9)	35.0	(7.0)	27.8	(6.6)	31.5	(4.9)	28.5	(5.66)	37.3	(6.0)	33.9	(7.2)
Anaemia (<30% hct)		N, %	40	17.5%	17	15.5%	205	62.7%	86	34.3%	87	41.2%	59	9.6%	407	26.6%
Geometric mean parasitaemia/uL			3331		18372		8864		29425		22788		9956		11247	
(range)			(40–173328)		(3768–156623)		(585–99502)		(32–200000)		(2080–192800)		(66–221433)		(32–221433)	
Mixed infection		N, %	n-a	n-a	n-a	n-a	40	12.2%	n-a	n-a	n-a	n-a	54	7.8%	n-a	
Gametocyte carriers		N, %	17	7.4%	1	0.9%	137	41.9%	8	3.2%	41	19.4%	33	4.8%	237	13.1%
Splenomegaly		N, %	54	23.6%	19	17.3%	n-a	n-a	25	10.0%	n-a	n-a	162	25.4%	260	21.2%
Hepatomegaly		N, %	24	10.5%	12	10.9%	n-a	n-a	1	0.4%	n-a	n-a	121	19.0%	158	12.9%
Fever (T.37.5C) on admission		N, %	153	67.1%	104	94.5%	148	45.4%	175	69.7%	211	100%	287	42.7%	867	54.6%

IQR; interquartile range.

n-a: no observation.

### Clinical recovery

Overall 54.6% of patients were febrile (≥37.5°C) on admission. This decreased to 8.8% at 24 h (i.e. median fever clearance <24 hours) and 2.1% at 48 h. No difference was detected between treatment groups. The median time for the spleen to be no longer palpable was 14 days (range 1–42). There was no difference in the time to resolution of splenomegaly between the treatment groups (P = 0.70).

### Parasitological efficacy

Of the 1,814 DP treated patients, 126 patients (6.9%) were lost to follow-up by Day 28. Of the 221 patients with recurrent infections, 9 cases had indeterminate PCR genotyping results, and 3 results were not available (lost samples). The results of parasitological efficacy per study site are shown in table 2, the number of patients followed-up, recurrent and recrudescent cases, and the daily results by category of follow-up are shown for a hypothetical cohort of 1000 persons with and without the PCR correction (table 3). On Day 1 (24 hours after starting treatment), 31% (95%CI 29–34) of the patients had cleared their parasitaemia, on Day 2, 89% (95%CI 87–90), on Day 3, 98% (95%CI 97–99). All patients had cleared their parasitaemia by Day 7.

**Table 2:** Dihydroartemisinin-piperaquine efficacy by country and site, Day 28, survival analysis (Kaplan-Meier).

Country and site		Efficacy (%) Day 28 *					
		PCR uncorrected			PCR corrected		
		Efficacy	Lower 95% confidence interval	Upper 95% confidence interval	Efficacy	Lower 95% confidence interval	Upper 95% confidence interval
Uganda, site Apac, Day 28		89.0	84.8	93.2	98.0	92.9	98.8
Rwanda all sites, Day 28		88.4	83.7	91.8	95.2	91.6	97.3
Rwanda site MA		94.2	86.5	96.6	96.6	89.5	98.9
Rwanda site KI		97.3	89.4	99.3	97.3	89.4	99.3
Rwanda site RU		75.2	64.4	89.7	89.7	82.7	95.8
Thailand all sites, Day 28		98.2	97.2	99.2	99.5	98.0	99.7
Thailand site KT		97.0	95.1	98.9	99.0	95.9	99.3
Thailand site MT		100.0	98.7	100.0	100.0	98.7	100.0
Thailand site TR		97.8	91.1	98.5	100.0	97.8	100.0
Cambodia all sites, Day 28		98.6	94.3	99.1	99.1	95.5	99.3
Cambodia site AV		99.0	95.3	99.3	99.0	95.3	99.3
Cambodia site KV		98.1	90.6	98.6	99.1	95.4	99.3
Myanmar all sites, Day 28		98.1	97.2	99.0	99.3	95.4	99.4
site MN		98.4	93.8	100.0	100.0	97.2	100.0
site DB		97.4	93.8	98.8	99.5	98.1	99.9
Laos, Day 28		100.0	96.6	100.0	100.0	96.6	100.0
Total, Day 28		96.1	95.0	97.2	98.7	97.6	99.8

* Efficacy based on randomised trials was assessed by modified intent to treat analysis for recurrent and recrudescent cases. The Kaplan-Meier results were expressed as percentages.

**Table 3:** Pooled efficacy of dihydroartemisinin-piperaquine against falciparum malaria based on randomised trials assessed by survival analysis (Kaplan-Meier).

Day group	Followed up	New infections			Recurrence (new infection+recrudescence)			Recrudescence		
	N	N	Quotient	Population free of recurrence *	N	Quotient	Population free of recurrence *	N	Quotient **	population free of recrudescence *
0	1814			100.0			100.0			100.0
7	1797	2	0.0011	99.9	2	0.0011	99.9		0.0000	100.0
14	1779	2	0.0011	99.8	3	0.0017	99.7	1	0.0006	99.9
21	1758	8	0.0046	99.3	12	0.0068	99.0	4	0.0023	99.8
28 ***	1736	35	0.0206	97.3	51	0.0298	96.1	16	0.0104	98.7
35	1453	42	0.0298	94.4	52	0.0364	92.6	10	0.0069	98.1
42 ****	1388	47	0.0350	91.1	49	0.0359	89.3	2	0.0014	98.0
49	799	10	0.0127	89.9	11	0.0138	88.0	1	0.0013	97.8
56	777	22	0.0291	87.3	23	0.0300	85.4	1	0.0013	97.7
63 *****	745	18	0.0248	85.1	18	0.0245	83.3	0	0.0000	97.7
Total		186			221			35		

* Pooled efficacy based on randomised trials was assessed by intent to treat analysis for recurrent and recrudescent cases. The Kaplan-Meier analysis was computed for an hypothetical cohort of 100 persons (the population free of disease per 100 is equivalent to efficacy expressed per 100). CI = confidence interval.

** Quotients adjusted for indeterminate cases.

*** Endpoint Rwanda.

**** Endpoint Laos, Uganda, Myanmar.

***** Endpoint Cambodia, Thailand.

### Primary endpoint: parasitological efficacy at Day 28

In DP groups, the overall observed parasitological efficacy using results from survival analysis at Day 28, corrected by PCR was 98.7% (95%CI 96.8–98.3). In children under 5 years old, the corresponding efficacy was lower: 94.2% (95%CI 91.9–96.5, P = 0.001). For the overall recurrence of *P. falciparum* parasitaemia the corresponding results were 96.1% (95%CI 95.0–97.2) and 90.4% (95%CI 87.8–93.0) in children (P = 0.001).

### Secondary endpoints

#### i) Risk of recrudescence for the full length of follow-up by treatment group

**Figure 2:**
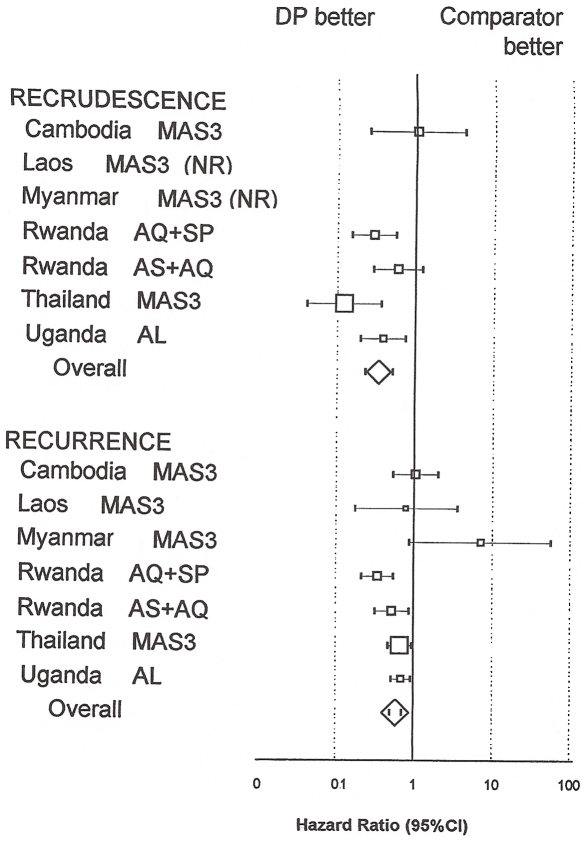
Note: HR; hazard ratio, CI; confidence interval. DP; dihydroartemisinin-piperaquine, MAS3; mefloquine-artesunate, AQ+SP; amodiaquine+sulfadoxine-pyrimethamine, AL; artemether-lumefantrine, AS+AQ; artesunate+amodiaquine. NC: not computable because of no recrudescent cases. *Overall number of failures does not add up because two comparators were used in Rwanda. Note: the forest plot represents the risk of parasite reappearance (PCR corrected; i.e. recrudescence, and not corrected i.e. recrudescence+new infection) of DP versus comparators in comparative studies. Groups size are equivalent except in Thailand where the DP group was twice as large (N = 686). Endpoints were assessed on Day 28 in Rwanda, Day 42 in Laos, Myanmar, and Uganda, and Day 63 in Cambodia, and Thailand. Overall results were stratified by site, and drugs. The size of the boxes is proportional to the number of patients included and thus to the overall effect. The diamond represents the overall hazard ratio and 95% CI.

Based on randomised comparisons by country and using the full length of follow-up of the different trials, DP recipients were at lower risk for a PCR confirmed failure compared to MAS3 in Thailand (P = 0.001), AL in Uganda (P = 0.004), and AQ+SP in Rwanda (P = 0.001)(figure 2, table S1). Overall, using multivariate analysis stratified by site and controlling for age, patients receiving DP had a lower risk of PCR confirmed treatment recrudescence than with the comparator treatments (AHR = 0.32, 95%CI 0.21–0.48, P = 0.001).

#### ii) Risk of recurrence for the full length of follow-up, by treatment group

Using multivariate analysis and the full length of follow-up stratified by site and controlling for age and anaemia, the overall risk of recurrence was lower in the DP groups than in the comparator groups (AHR = 0.60, 95%CI 0.51–89, P = 0.001)(figure 2). DP provided a better protective effect against *P. falciparum* recurrence than its comparator groups in Rwanda; compared to AQ+SP (P = 0.006) and AS+AQ (P = 0.006); to AL in Uganda (P = 0.005); and MAS3 in Thailand (P = 0.016).

There was a longer interval from primary infection to recurrence (suggesting a greater duration of suppressive prophylaxis) compared to MAS3 in Thailand (median: 49 days vs. 37 days, respectively, P = 0.001); in Uganda compared to AL (median 35 vs. 28 days, respectively, P = 0.001); in Rwanda compared to AQ+SP (median 28 vs. 21 days, respectively, P = 0.035), but was not different to AS+AQ (P = 0.23).

#### iii) Risk of new infections (PCR confirmed) by Day 28

At 28 days the risk of new infection was greater in African compared to Asian settings (P = 0.001) reflecting the higher transmission intensity. In these high transmission areas, patients treated with DP were at lower risk for a new infection within 28 days compared to AQ+SP (HR = 0.38, 95%CI 0.19–0.76, P = 0.006), AS+AQ (HR = 0.42, 95%CI 0.21–0.86, P = 0.018) in Rwanda and AL (HR = 0.38, 95%CI 0.22–0.65, P = 0.001) in Uganda. Overall, in the multivariate analysis stratified by site, DP had a greater post treatment prophylactic effect (against new infections) at Day 28 against *P. falciparum* compared to the other treatments (AHR = 0.47, 95%CI 0.33–0.67, P = 0.001).

#### iv) Predictors of recrudescence, new infection, and recurrence of *P. falciparum* in DP groups by Day 28

In the DP groups, using multivariate analysis stratified by site at Day 28, age (as continuous variable) was the only predictor of recurrence and recrudescence in Africa or Asia when analyzed separately. In African children, younger patients (per 1 year increase in age) were at higher risks for recurrence (AHR = 0.86, 95%CI 0.77–96, P = 0.006), and recrudescence (AHR = 0.80, 95%CI 0.68–96, P = 0.018), as well as in Asian patients (AHR = 0.93, 95%CI 0.90–0.96, P = 0.001; and AHR = 0.81, 95%CI 0.73–0.89, P = 0.001, respectively).

No significant predictors of new infections were detected in African children, but in Asian patients younger patients were at higher risks for new infections (AHR = 0.96, 95%CI 0.93–0.99, P = 0.025).

#### v) Gametocyte carriage

Admission pre-treatment gametocytaemia was present in a median (range) of 6.1% (0.9–41.9) of the patients. Using multivariate analysis in DP groups, and controlling by site, younger patients, admission anaemia, and a lower admission parasite count were related to a higher risk of patent gametocytaemia (table 4).

**Table 4:** Predictors of patent gametocytaemia, and gametocyte appearance, dihydroartemisinin-piperaquine groups.

Independent variables	Gametocyte on admission	Gametocyte appearance
Age continuous	AOR = 0.97, 95%CI 0.95–0.98, p = 0.001	
Anaemia (ref: no anaemia)	AOR = 1.83, 95%CI 1.43–2.34, p = 0.001	
Parasite count (continuous)	AOR = 0.69, 95%CI 0.58–0.82, p = 0.001	
Model 1 for recurrence		
Anemia on admission		AHR = 2.71, 95%CI 1.56–4.73, p = 0.001
Recurrence during follow-up		AHR = 2.66, 95%CI 1.32–5.39, p = 0.001
Model 2 for recrudescence		
Anemia on admission		AHR = 2.83, 95%CI 2.61–8.66, p = 0.001
Recrudescence during follow-up		AHR = 2.90, 95%CI 0.68–12.45, p = 0.152

Note: Age was per 1 year increase in age, and parasite count was per 1 unit increase in the log transformed parasite density. AOR; adjusted odds ratio. AHR; adjusted hazard ratio. CI; confidence interval.

**Figure 3:**
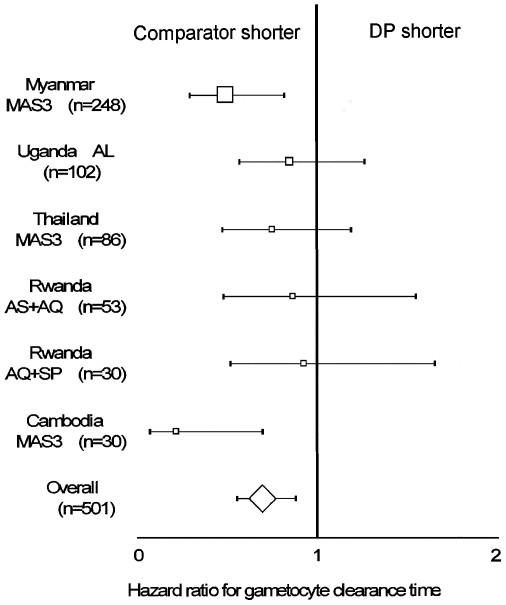
Note: the forest plot represents the risk of clearing gametocytes comparing DP versus comparator drugs in randomised studies. The endpoint was assessed at Day 14. Overall result was stratified by site. The size of boxes is proportional to the number of patients included and thus to the overall effect. The diamond represents the overall hazard ratio and 95% CI.

Clearance of gametocytaemia was slower in DP groups than in the comparators, overall and in individual sites. In Cambodia in the DP treatment arm, 82(95%CI 55–94)% of patients presenting with gametocytaemia still had gametocytaemia on Day 3 compared to only 27(7–54)% in the comparator arm. On Day 14 of the follow-up in Thailand 24(10–39)% in DP arm and 11(2–30)% in the other arm, in Uganda 7.4(2.4–16)% in DP arm compared to 1.8(0.3–13)% in the other arm, in Myanmar 31(23–38)% in DP arm as compared to none in the comparator arm. Overall, using multivariate analysis, the risk of gametocyte carriage by Day 14 was lower in comparators than in DP groups (P = 0.002, stratified by site)(figure 3, table S2).

**Figure 4:**
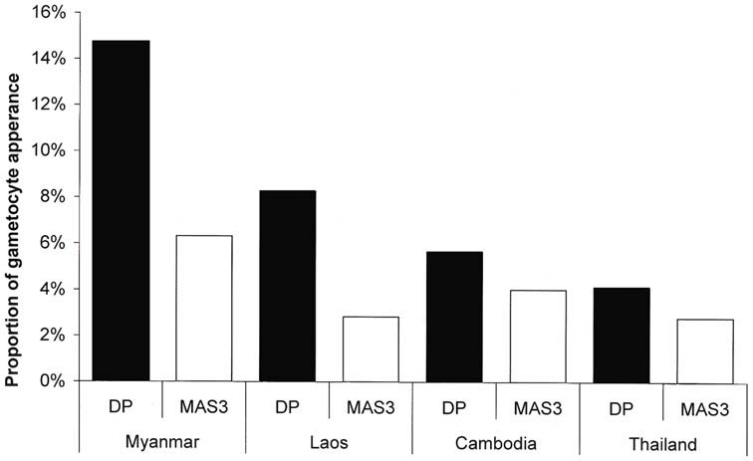
Note: DP; Dihydroartemisinin-piperaquine, MAS3; mefloquine-artesunate.

Overall 178 (8.4%) out of 2125 patients presenting without gametocytes developed gametocytaemia after starting treatment. Recurrent parasitaemia as well as anaemia on admission were associated with gametocyte appearance (Table 4). There were no significant overall differences in the risk of developing gametocytaemia during follow-up between DP and all comparator arms (Mantel-Haenszel OR = 1.02 [95%CI 0.75–1.40], P = 0.89; P = 0.035 for homogeneity between studies). However, there were significant differences in appearance of gametocytes in 3 Asian countries (Cambodia, Myanmar, Thailand) between DP and MAS3 (Mantel-Haenszel OR = 1.89, 95%CI 1.23–2.91, P = 0.003; P = 0.643 for homogeneity between studies). Including the Laos results, which only recorded if the patients had gametocyte after admission as a sole binary variable, the proportion of patients with gametocyte appearance within 42 days was higher in DP groups (6.5%, 76/1164) compared to MAS3 groups (4.0%, 35/873) (OR = 1.96, 95%CI 1.28–3.09, P = 0.002, Mantel-Haenszel weighted by site)(figure 4).

In Cambodia, Myanmar, and Thailand, the overall gametocyte carriage rate within 42 days was greater in the DP (56/1000 PGW, 260/4675, 95%CI 49–62) compared to MAS3 groups (36/1000 PGW, 100/2772, 95%CI 29–43)(RR = 1.36, 95%CI 1.06–1.76, P = 0.017, Mantel-Haenszel weighted by site). In patients without gametocytaemia on admission and who developed gametocytaemia during the follow-up, the carriage rate was also greater with DP (11/1000 PGW) than MAS3 (6/1000 PGW)(RR = 2.88, 95%CI 1.51–6.28, P = 0.001, Mantel-Haenszel weighted by site).

When using available data (from Thailand, Cambodia, Myanmar, and Uganda) there was no relationship between the dose of DHA actually received by the patient and the proportion of patients remaining with gametocytaemia on Day 14 (P = 0.382).

#### vi) Haematological changes

On admission, 537 out of 1,797 (29.9%) DP recipients with available data were anaemic (Hct<30%). Of these 29.8% (74/537) had severe anaemia (Hct<20%). Using multivariate analysis, anaemia on admission was strongly associated with age and varied by country (Table 1). Children under 15 y were at higher risk for anaemia compared to adults as well as patients from Cambodia, Uganda, and Myanmar compared to Thailand (P = 0.001, for all comparisons). By Day 28, 55% (162/293) of the anaemic patients had recovered from anemia and 2.8% (24/851) who were not anaemic on admission became anaemic. Overall, in anaemic patients, the median time to recovery (defined as haematocrit ≥30%) ranged from 7 to 42 days. On Day 42, the prevalence of anaemia was 3.4% (34/985). There were no treatment differences in the development of anaemia.

In Cambodia, Laos, Myanmar, Thailand, there was a relative mean paired transient decline of 6.3% (95%CI 5.7–7.3) in haematocrit from admission to Day 7 in DP groups. No difference was detected compared to the MAS3 group. By contrast, in Rwanda, between Day 0 and Day 14, the relative mean paired haematocrit difference was significantly higher in the AS+AQ group (+10%, 95%CI 8–11) compared to the DP group (+6%, 95%CI 4–8)(P = 0.021). In Uganda, patients treated with DP (+20%, 95%CI 16–24) had a higher relative paired mean increase in haemoglobin levels on Day 42 compared to AL group (+16%, 95%CI 12–20, P = 0.049).

#### vii) Drug vomiting, incidence of adverse events, number of adverse events, and death

Overall, vomiting on admission and before treatment administration was a risk factor for vomiting the first dose of DP (OR = 6.1, 95%CI 3.2–11.7) and for vomiting DP treatment over the three days (OR = 4.6, 95%CI 2.7–7.7). This was similar in every country. In patients who did not present with vomiting on admission, the overall incidence of early vomiting (defined as vomiting the drug within 1 hour after intake) DP was low; 1.7% (21/1,231) on Day 0. Over the 3 days of treatment, the overall incidence rate of early vomiting ranged from 3.2 (95%CI 2.1–4.8)% in Thailand to 9.9 (95%CI 6.8–14.4)% in Rwanda (Table 5). In DP groups, drug vomiting was more frequent in Rwanda than all other countries (P = 0.001) and was related to age: the 0–4 y age group (OR = 8.2, 95%CI 3.2–21.3), and the 5–14 y age group (OR = 3.9, 95%CI 1.9–8.1) were at higher risk compared to adults. In Rwanda, the incidence of overall vomiting DP was not different to that after AS+AQ (11.5%, P = 0.565), but the risk was much lower than with AQ+SP (19.5%, P = 0.002). No difference in the incidence of early vomiting after drug treatment was observed between DP and MAS3 on Day 0 (3.2%, and 2.4%, respectively, P = 0.400), or overall (3.7%, and 4.2%, respectively, P = 0.671).

**Table 5:** Early vomiting (<one hour) after treatment administration, dihydroartemisinin-piperaquine groups.

Country	Daily incidence			Total incidence	95% confidence interval	N
	Day 0	Day 1	Day 2			
Myanmar	2.1%	1.2%	0.6%	4.0%	2.3%–6.8%	13/327
Cambodia	4.8%	1.3%	0.0%	5.2%	3.0%–9.1%	12/228
Rwanda	6.7%	2.4%	2.8%	9.9%	6.8%–14.4%	25/252
Thailand	2.6%	0.6%	0.1%	3.2%	2.1%–4.8%	22/686
Total	3.5%	1.1%	0.5%	4.8%	3.7%–5.9%	72/1495

We examined the incidence and prevalence of 24 other different adverse events (apart from early vomiting) in 1,267 individuals using available records from DP groups in five countries and 9 different sites. It was not possible to calculate the adverse events duration in Laos, and Uganda. In the remaining DP groups, the five most commonly reported adverse events by Day 28 were 23.3% for headache, 17.0% for dizziness, 13.8% for sleep disturbance, 11.6% for anorexia, 10.5% for nausea (table 6). Hypersensitivity reactions including urticaria were reported in 4 patients in Thailand (0.6%, 4/686, 95%CI 0.2–1.5) [6]. The following adverse events: muscle pain, hearing disturbances, itching, nightmares, visual disturbances, dyspnoea, numbness, skin rash, agitation and confusion, were all reported in less than 5% of cases. The maximum point prevalence rates of the adverse events all occurred on Day 1. Day 1 and Day 2 captured 54% and the first week captured 70% of the reported adverse event incidence.

**Table 6:** Adverse event incidence density and prevalence rates, dihydroartemisinin-piperaquine groups.

Adverse event	Adverse event cumulative incidence density over 28 days				Incidence within the first week*	Adverse event maximum prevalence			
	Number of patients without the symptom on admission	%	Lower 95%CI	Upper 95%CI		Day	%	Lower 95%CI	Upper 95%CI
Headache	124	23.3%	15.4%	31.2%	74%	Day 1	9.9%	5.8%	17.0%
Dizziness	457	19.3%	15.3%	23.2%	88%	Day 1	11.6%	8.5%	14.8%
Sleeping problem	811	18.4%	15.0%	21.7%	83%	Day 1	6.3%	4.8%	8.2%
Anorexia	521	16.7%	13.4%	19.9%	85%	Day 1	10.3%	7.7%	13.0%
Fatigue	628	15.0%	12.4%	18.1%	82%	Day 1	7.7%	5.9%	10.1%
Nausea	628	13.6%	10.8%	16.5%	83%	Day 1	8.7%	6.4%	11.0%
Joint pain	451	10.0%	7.6%	13.3%	63%	Day 1	4.7%	3.1%	7.2%
Abdominal pain	1040	9.6%	7.8%	11.4%	76%	Day 1	4.7%	3.4%	6.0%
Diarrhoea	1204	9.2%	7.5%	10.9%	84%	Day 1	4.8%	3.6%	6.1%
Late vomiting	1083	7.1%	5.5%	8.7%	86%	Day 1	4.7%	3.4%	6.0%
Palpitations	753	6.7%	5.1%	8.8%	82%	Day 1	3.2%	2.2%	4.8%
Hearing disturbance	971	6.3%	4.5%	8.1%	68%	Day 1	2.4%	1.3%	3.5%

CI; confidence interval.

* The incidence within the first week is the proportion of cases occurring in the first week divided by the total number of cases within 28 days for each adverse event.

**Figure 5:**
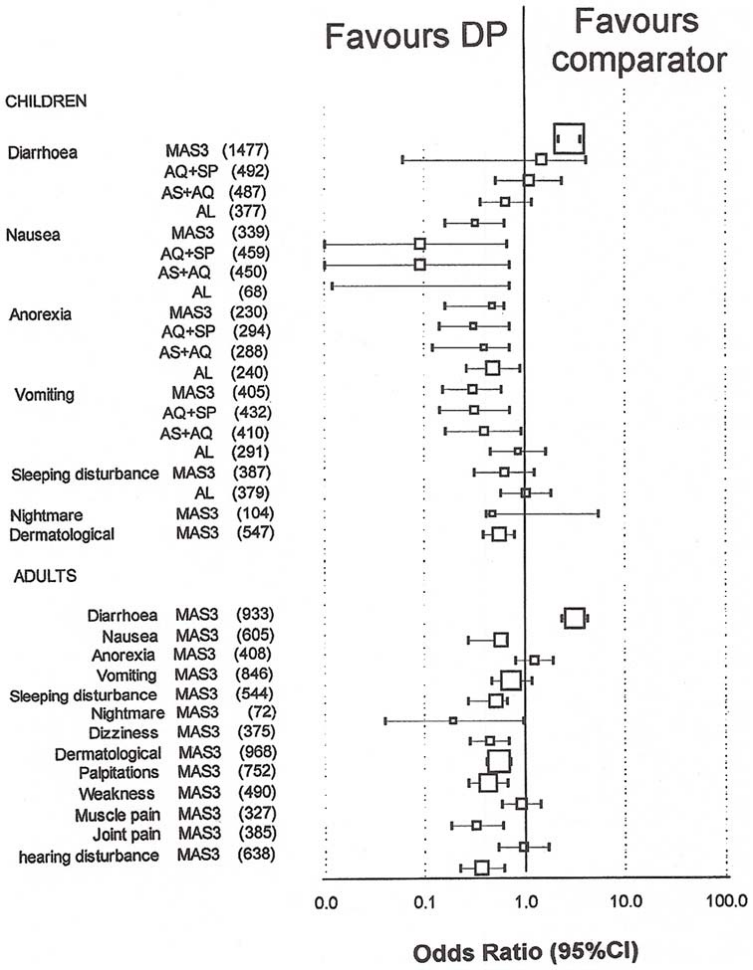
Note: the forest plot represents the risk of adverse event appearance after the start of dihydroartemisinin-piperaquine treatment in children (<15 y) and adults who did not present this symptom on admission versus comparators in comparative studies. The size of boxes is proportional to the number of patients included. 95% confidence intervals (CI) are calculated for the odds ratio (OR).

In the Asian trials, the only adverse event that was significantly more frequent in DP treated patients compared to the MAS3 group was diarrhoea in all age groups (children: 36% 310/852 vs. 17% 108/625, OR = 2.74, 95%CI 2.13–3.51; adults: 46% 248/543 vs. 21% 83/390, OR = 3.11, 95%CI 2.31–4.18, respectively, P = 0.001 for both comparisons, figure 5, table S3). Regarding other gastro-intestinal adverse events, DP was significantly better tolerated than AS+AQ, AS+SP, or MAS3 (P<0.040 for all comparisons) but was not different compared to AL (only for late vomiting). In adults the risk of nightmare (P = 0.028) and sleep disturbances (P = 0.003) was lower in the DP group than in the MAS3 group. The risk of dermatological events (P = 0.002), dizziness, palpitation, and muscle pain in all ages was also lower than in the MAS3 group (P<0.010 for all comparisons). The risks of hearing disturbance (tinnitus, or hearing problems) in adults treated with MAS3 was greater than in the DP group (P = 0.001).

The frequency of patients treated with DP and reporting at least one of the 24 adverse events analyzed was 57.2% (840/1,468, 95%CI 54.7–59.8); among them 38% reported one, and 26% two adverse events (without excluding patients under 5). The total number of adverse events reported per patient was higher in older patients (P = 0.001) and in anaemic patients on admission (P = 0.020 after correcting for age).

The incidence of adverse events was significantly lower in DP recipients compared to MAS3 recipients (on average by −59%, 95%CI −28 to −90%, P = 0.001). More patients treated with MAS3 reported two or more AEs (67%, 454/675, P = 0.034). In Rwanda, the risk of having any adverse event was higher in the AQ+SP group (OR = 2.19, 95%CI 1.35–3.57) and in the AS+AQ group (OR = 1.90, 95%CI 1.15–3.12) compared to the DP group. No differences were detected in Uganda in the AL group compared to the DP group.

A child from Rwanda had a seizure and received a rescue treatment. Overall, 5 deaths occurred in DP groups, all of which were considered to be unrelated to the treatment, except in Thailand where a 43-year-old woman who died from severe malaria the day after entering the study, which might have been related to a lack of efficacy of the drug. In Thailand, there were another 2 deaths: a 13-year-old girl who died on Day 7 from probable bacterial sepsis, and a 21 year old male who died on Day 28 from gunshot wounds. In Laos, a two year old child died on Day 37 from cerebral malaria after probable reinfection. In Myanmar, one 11-year-old child died after developing fever on Day 20 and had generalized seizures the next morning (Day 21). The malaria smear was negative. In the comparator groups, one death occurred in MAS3 groups in Thailand involving a malaria-smear negative 13-year-old boy, who was clinically well by the third day of treatment. He was reported to have deteriorated rapidly with worsening abdominal pain and distension, jaundice, and anuria, and he died within a few hours.

#### viii) Plasmodium vivax and other species appearing during the follow-up period

**Figure 6:**
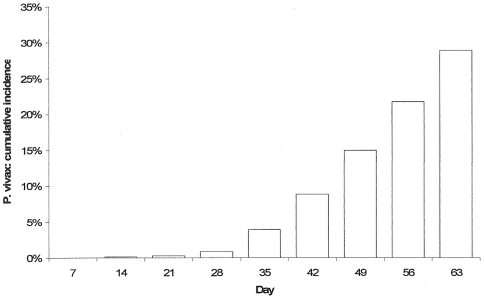
P.vivax cumulative incidence density, dyhydroartemisinin-piperaquine treatment groups. Patients receiving DP in Cambodia, Laos, Myanmar, and Thailand who had *P. vivax* parasitaemia detected during the follow-up.

No difference was detected between DP groups and the comparator (mefloquine-artesunate) in *P. vivax* recurrence rates (P>0.05 for all comparisons). In Cambodia, Laos, Myanmar, and Thailand, 265 patients receiving DP (28.8/100 person-days within 63 days) had *P. vivax* parasitaemia detected during the follow-up (figure 6). The median (range) time to the *P. vivax* parasitaemia was 49 (14–63) days compared with 49 (28–63) days, in MAS3 groups. Patients with *P. vivax* on admission (mixed infections) were at higher risk of having a second *P. vivax* episode during the follow-up (HR = 1.70, 95%CI 1.15–2.51, P = 0.008). Children were at increased risk of *P. vivax* recurrence when compared to adults; 0–4 age group (HR = 2.85, 95%CI 1.81–4.48, P = 0.001) and the 5–14 age group (HR = 1.43, 95%CI 1.05–1.94, P = 0.024). In patients who had mixed infections on admission, the median time to *P. vivax* recurrence was 42 days (7–63), significantly shorter than in patients presenting with a *P. falciparum* monoinfection: 49 (16–70) days, (P = 0.029). In Uganda patients treated with DP had a lower risk of recurrent parasitaemia due to *P. malariae* and *P. ovale* species compared to patients treated with AL (5.2% versus 0.9%, P = 0.001)[10].

## Discussion

Since initial deployment in 1994 of the mefloquine-artesunate (MAS3) combination to treat *P. falciparum* malaria along the Thai-Myanmar border, there has been increasing use of ACTs throughout the malaria affected world. Dihydroartemisinin-piperaquine (DP) is a relatively new and very promising fixed dose ACT which has been extensively evaluated in the past few years. This analysis of 3,547 patients (1,814 of whom received DP) in randomized comparative clinical trials includes 7 of the 22 published studies (until December 2008), but is broadly representative as it comprises a wide patient age range and derives from areas of widely differing intensity of malaria transmission. It is the first individual patient data set compiled prospectively, based on randomized comparisons in studies of generally similar overall design. It includes half (49%, 1,814/3,678) of all the patients treated with DP in published clinical trials with parasite genotyping corrected results and reports 43% (1,814/4,212) of all patients included in published studies to date.

To present the results of this analysis of individual patient data we have used a method similar to that used for a meta-analysis of trials (for instance in Cochrane's review) with graphical representation of risks, recommended for communicating in medical research [22]. Compared to a “conventional” meta-analysis from published studies, the analysis of individual patient level data increases statistical power by facilitating analytical practice, and enables standardized estimates of drug efficacy across different studies.

In the 12 different African and Asian sites with varying levels of background antimalarial drug resistance, DP was well tolerated and highly efficacious. Like all ACTs DP produces rapid therapeutic responses with swift resolution of symptoms and fever and clearance of parasitaemia. Efficacy (PCR corrected) exceeded 96% in all sites except one in Rwanda, where no food was co-administered with the drug. Treatment with DP was associated with lower risks of overall recurrence (P = 0.001), recrudescence (P = 0.001), and new infections (P = 0.001) compared with the comparator drugs. The superiority of DP was demonstrated in Rwanda, Uganda and Thailand against the local current first-line treatments. This excellent efficacy and tolerability profile suggests that DP could be considered as a potential alternative first-line antimalarial treatment. This result based on studies of 1,814 adults and children who received antimalarial treatment with DP, contrasts with the recently reported finding of a study of 100 young children in Papua New Guinea [23]. DP was not administered with food in that study, a factor that could contribute to lower oral piperaquine absorption, and reduce therapeutic efficacy [24]. High levels of chloroquine resistance were considered a possible explanatory factor, although levels of chloroquine resistance were also very high in the study sites in this multi-center analysis. PCR corrected efficacy in adjacent Papua with DP treatment was over 95%, although this trial enrolled adults and children [25]. The reasons for lower efficacy in Rukara, Rwanda are unclear but consistent with the lower efficacy of other ACTs such as amodiaquine-artesunate at this site compared to other Rwandan sites [26] and could also be related with the fact that DP was not administered with food. The superior protective effect of DP against reinfection and suppression of vivax relapses compared with the other drugs presumably results from the long terminal half-life of piperaquine. Suppression of reinfection provides longer disease free intervals but at the expense of increased selection pressure for resistance to piperaquine compared with more rapidly eliminated partner drugs.

DP was less potent than mefloquine–artesunate in suppressing gametocytaemia. Similar results were also observed in Kenya in comparison with AL [27], and in Peru compared to MAS3 [28]. Taken together these results indicate that the reduction in gametocytaemia, an important pharmacodynamic advantage of ACTs, is less pronounced with DP than with other ACTs. This could be related to the relatively lower dose of DHA (2.5 mg/kg/day) compared to the dose of artesunate in the comparators (4 mg/kg/day). But in our analysis we found no relationship between the dose of DHA received by the patients in the DP arms and the clearance of gametocytaemia. Prolonged gametocytemia has been proposed as an early sign of the emergence of drug resistance [29]. This might be a concern given the poorer gametocytocidal effects of DP. However the gametocyte carriage rate remained low, and the gametocyte clearance was fast. A relatively few patients had detectable gametocytemia during the studies, which reflects the potent gametocytocidal properties of the artemisinin derivatives.

DP was effective in treating non-*P. falciparum* species co-infections. Unlike *P. falciparum* infections, recurrence of *P. vivax* (either relapses or failures) cannot be reliably distinguished from a new infection [30]. Mixed infections are common. Approximately one third of acute falciparum malaria infections in the South-East Asian region are followed by a vivax malaria relapse. In trials with 63 days follow-up the recurrent episode of vivax malaria in patients with mixed infections on admission occurred slightly earlier (6 weeks) than new vivax appearances in patients with a falciparum mono-infection (7 weeks). Thus, the first relapse is suppressed by DP, comparable to the effect of chloroquine on sensitive *P. vivax* strains [31].

The higher risk of treatment failure in children treated with DP compared with adults is similar to the pattern seen with other antimalarial drugs, and presumably results both from lower immunity and lower blood piperaquine concentrations. The shorter time to *P. vivax* reappearance in children would also support a pharmacokinetic explanation. The Day 7 piperaquine level is a useful measure of drug exposure. Young children have lower piperaquine levels on Day 7 and higher treatment failure rates than older children and adults [4], [25]. In a recently reported population pharmacokinetic study from Thailand, there were therapeutically relevant pharmacokinetic differences between different age groups. Children had a smaller central volume of distribution, a shorter distribution half-life (t_1/2, α_), and a more rapid fall in initial PQ plasma concentrations compared to the population mean profile [4]. Studies indicated lower plasma piperaquine concentration in children compared to adults in Papua, Indonesia [25] and higher clearance in children in Vietnam [32]. Taken together these data argue for higher weight adjusted doses in children compared with adults. This would also have the advantage of providing a larger dose of dihydroartemisinin. Further studies in young children to optimize the dose of DP are needed.

The risk of early vomiting of DP was lower than following AQ+SP in Rwanda (P = 0.001). No differences were observed with the other comparator treatments. Early vomiting was more frequent in young children, although it was not a risk factor for treatment failure, presumably because these children were re-dosed successfully after vomiting the drug.

The DP safety profile has been excellent in all published series. The overall safety analysis showed that the risk of the most common adverse events was significantly lower following DP treatment than in the comparator arms in both children (<15 years old) and adults. Adverse events were often related to the disease itself (particularly neurological and gastro-intestinal AEs), although diarrhea was approximately twice as common following DP than with MAS3.

The use of common protocols for data collection to assess antimalarial drug efficacy and tolerability allows combination of these data into larger international databases which can give us more information on the safety and efficacy of these drugs in different patient groups [33]. While methods for assessing antimalarial drug efficacy are well standardized there is little uniformity in safety reporting in antimalarial drug studies, a problem which needs to be addressed.

DP is not yet recognized internationally and its use has been limited by its regulatory status. The formulation used in the trials was donated by Holley and manufactured according to Chinese SFDA Good Manufacturing Practice (GMP) standards. Nevertheless, differences in efficacy might be related to the variability in the composition of the study drug.

This antimalarial combination is currently under evaluation by the WHO pre-qualification process. DP is clearly an important new antimalarial drug. It is well tolerated, highly effective and safe. The higher rates of gametocytaemia compared with other ACTs and lower piperaquine levels early in the terminal elimination phase observed in children suggest that dosage may have to be increased in this important patient group.

## Supporting Information

Table S1 (for figure 2): Recurrences (PCR uncorrected) and recrudescences (PCR corrected) comparing the risks in the dihydroartemisinin-piperaquine group versus the comparator arms by drug and country of study. *Overall number of failures does not add up because two comparators were used in Rwanda. Note: the forest plot represents the risk of parasite reappearance (PCR corrected; i.e. recrudescence, and not corrected i.e. recrudescence+novel infection) of DP versus comparators in comparative studies. Groups size are equivalent except in Thailand where the DP group was twice as large (N = 686). Endpoints were assessed on Day 28 in Rwanda, Day 42 in Laos, Myanmar, and Uganda, and Day 63 in Cambodia, and Thailand. Overall results were stratified by site, and drugs. HR: hazard ratio
(0.07 MB DOC)

Table S2 (for figure 3): Risks of clearing gametocytaemia by Day 14 in patients with gametocytaemia on admission, dihydroartemisinin-piperaquine (DP) group versus comparators arms by drug and country of study. HR; hazard ratio, CI; confidence interval. (NC): not computable because of the day of clearance not available.
(0.05 MB DOC)

Table S3 (for figure 5): Day 28 adverse event risks for ‘treatment’ DP versus controls (comparators)Note: The risk of adverse event appearance after the start of dihydroartemisinin-piperaquine treatment in children (<15 y) and adults who did not present this symptom on admission versus comparators in comparative studies. 95% confidence intervals (CI) are calculated for the odds ratio (OR)
(0.12 MB DOC)
