# Supplementary material for: Safety and Efficacy of Dihydroartemisinin-Piperaquine in Falciparum Malaria: A Prospective Multi-Centre Individual Patient Data Analysis
Source: PLoS One. 2009 Jul 29;4(7):e6358. doi: 10.1371/journal.pone.0006358 (PMC2716525; doi:10.1371/journal.pone.0006358)
Supplement: Table S1 — (for figure 2): Recurrences (PCR uncorrected) and recrudescences (PCR corrected) comparing the risks in the dihydroartemisinin-piperaquine group versus the comparator arms by drug and country of study. *Overall number of failures does not add up because two comparators were used in Rwanda. Note: the forest plot represents the risk of parasite reappearance (PCR corrected; i.e. recrudescence, and not corrected i.e. recrudescence+novel infection) of DP versus comparators in comparative studies. Groups size are equivalent except in Thailand where the DP group was twice as large (N = 686). Endpoints were assessed on Day 28 in Rwanda, Day 42 in Laos, Myanmar, and Uganda, and Day 63 in Cambodia, and Thailand. Overall results were stratified by site, and drugs. HR: hazard ratio (0.07 MB DOC) [file pone.0006358.s001.doc]

Supporting information 1 (for figure 2): Recurrences (PCR uncorrected) and recrudescences (PCR corrected) comparing the risks in the dihydroartemisinin-piperaquine group versus the comparator arms by drug and country of study

| Comparator | | HR | Lower 95%CI | Upper 95%CI | P | N failure* | |
| --- | --- | --- | --- | --- | --- | --- | --- |
|  |  |  |  |  |  | DP | Comparator |
| PCR corrected |  |  |  |  |  |  |  |
| Cambodia | MAS3 | 1.00 | 0.25 | 4.02 | 0.994 | 4 | 4 |
| Laos | MAS3 | NC |  |  |  | 0 | 0 |
| Myanmar | MAS3 | NC |  |  |  | 2 | 0 |
| Rwanda | AQ+SP | 0.29 | 0.15 | 0.55 | 0.001 | 12 | 39 |
| Rwanda | AS+AQ | 0.58 | 0.29 | 1.19 | 0.140 | 12 | 20 |
| Thailand | MAS3 | 0.12 | 0.04 | 0.36 | 0.001 | 4 | 16 |
| Uganda | AL | 0.38 | 0.20 | 0.74 | 0.004 | 13 | 28 |
| Overall* |  | 0.32 | 0.21 | 0.48 | 0.001 | 35 | 107 |
|  |  |  |  |  |  |  |  |
| PCR not corrected | |  |  |  |  |  |  |
| Cambodia | MAS3 | 1.01 | 0.53 | 1.94 | 0.975 | 22 | 22 |
| Laos | MAS3 | 0.77 | 0.17 | 3.42 | 0.726 | 3 | 4 |
| Myanmar | MAS3 | 5.94 | 0.72 | 49.31 | 0.099 | 6 | 1 |
| Rwanda | AQ+SP | 0.38 | 0.19 | 0.76 | 0.006 | 24 | 66 |
| Rwanda | AS+AQ | 0.52 | 0.31 | 0.85 | 0.009 | 24 | 45 |
| Thailand | MAS3 | 0.84 | 0.46 | 0.92 | 0.016 | 76 | 55 |
| Uganda | AL | 0.67 | 0.51 | 0.89 | 0.005 | 90 | 108 |
| Overall |  | 0.58 | 0.49 | 0.69 | 0.001 | 221 | 301 |

*Overall number of failures does not add up because two comparators were used in Rwanda. Note: the forest plot represents the risk of parasite reappearance (PCR corrected ; i.e. recrudescence, and not corrected i.e. recrudescence + novel infection) of DP versus comparators in comparative studies. Groups size are equivalent except in Thailand where the DP group was twice as large (N=686). Endpoints were assessed on Day 28 in Rwanda, Day 42 in Laos, Myanmar, and Uganda, and Day 63 in Cambodia, and Thailand. Overall results were stratified by site, and drugs. HR: hazard ratio
