# Supplementary material for: Safety and Efficacy of Dihydroartemisinin-Piperaquine in Falciparum Malaria: A Prospective Multi-Centre Individual Patient Data Analysis
Source: PLoS One. 2009 Jul 29;4(7):e6358. doi: 10.1371/journal.pone.0006358 (PMC2716525; doi:10.1371/journal.pone.0006358)
Supplement: Table S2 — (for figure 3): Risks of clearing gametocytaemia by Day 14 in patients with gametocytaemia on admission, dihydroartemisinin-piperaquine (DP) group versus comparators arms by drug and country of study. HR; hazard ratio, CI; confidence interval. (NC): not computable because of the day of clearance not available. (0.05 MB DOC) [file pone.0006358.s002.doc]

| Supporting information 2 (for figure 3): Risks of clearing gametocytaemia by Day 14 in patients with gametocytaemia on admission, dihydroartemisinin-piperaquine (DP) group versus comparators arms by drug and country of study   | Comparator | | HR | Lower 95%CI | Upper 95%CI | P | N cleared | | | --- | --- | --- | --- | --- | --- | --- | --- | | DP | Comparator | | Gametocytaemia  cleared |  |  |  |  |  |  |  | | Myanmar | MAS3 | 0.48 | 0.29 | 0.81 | 0.006 | 4 | 9 | | Uganda | AL | 0.86 | 0.56 | 1.26 | 0.844 | 42 | 55 | | Thailand | MAS3 | 0.75 | 0.47 | 1.19 | 0.220 | 44 | 29 | | Rwanda | AQ+SP | 0.93 | 0.52 | 1.66 | 0.795 | 19 | 28 | | Rwanda | AS+AQ | 0.86 | 0.48 | 1.55 | 0.616 | 19 | 26 | | Laos | MAS3 | NC |  |  |  |  |  | | Cambodia | MAS3 | 0.21 | 0.06 | 0.69 | 0.010 | 4 | 9 | | Overall |  | 0.70 | 0.55 | 0.88 | 0.002 | 113 | 156 | |  |
| --- | --- | --- | --- | --- | --- | --- | --- | --- | --- | --- | --- | --- | --- | --- | --- | --- | --- | --- | --- | --- | --- | --- | --- | --- | --- | --- | --- | --- | --- | --- | --- | --- | --- | --- | --- | --- | --- | --- | --- | --- | --- | --- | --- | --- | --- | --- | --- | --- | --- | --- | --- | --- | --- | --- | --- | --- | --- | --- | --- | --- | --- | --- | --- | --- | --- | --- | --- | --- | --- | --- | --- | --- | --- | --- | --- | --- | --- | --- | --- | --- | --- | --- | --- |

HR; hazard ratio, CI; confidence interval. (NC): not computable because of the day of clearance not available.
