# Supplementary material for: Safety and Efficacy of Dihydroartemisinin-Piperaquine in Falciparum Malaria: A Prospective Multi-Centre Individual Patient Data Analysis
Source: PLoS One. 2009 Jul 29;4(7):e6358. doi: 10.1371/journal.pone.0006358 (PMC2716525; doi:10.1371/journal.pone.0006358)
Supplement: Table S3 — (for figure 5): Day 28 adverse event risks for ‘treatment’ DP versus controls (comparators)Note: The risk of adverse event appearance after the start of dihydroartemisinin-piperaquine treatment in children (<15 y) and adults who did not present this symptom on admission versus comparators in comparative studies. 95% confidence intervals (CI) are calculated for the odds ratio (OR) (0.12 MB DOC) [file pone.0006358.s003.doc]

## **Supporting information 3 (for figure 5): Day 28 adverse event risks for ‘treatment’ DP versus controls (comparators)**

| | | **Adverse event** | **Treatment** | **OR** | **Lower 95%CI** | **Upper 95%CI** | **P** | | **Number of  adverse event** | | | | --- | --- | --- | --- | --- | --- | --- | --- | --- | --- | |  |  |  |  |  |  |  | **DP** |  | **Comparator** | | **CHILDREN** |  |  |  |  |  |  |  |  |  | | **Diarrhoea** | MAS3 | 2.74 | 2.13 | 3.51 | 0.000 | * | 310 | v | 108 | |  | AQ+SP | 1.44 | 0.06 | 4.03 | 0.373 |  | 15 | v | 11 | |  | AQ+AS3 | 1.09 | 0.51 | 2.31 | 0.821 |  | 15 | v | 14 | |  | AL | 0.64 | 0.36 | 1.15 | 0.134 |  | 22 | v | 33 | | **Nausea** | MAS3 | 0.37 | 0.16 | 0.62 | 0.000 | * | 15 | v | 29 | |  | AQ+SP | 0.09 | 0.01 | 0.66 | 0.003 | * | 1 | v | 12 | |  | AQ+AS3 | 0.09 | 0.01 | 0.70 | 0.004 | * | 1 | v | 11 | |  | AL | - | 0.01 | 0.70 | 0.138 |  | 0 | v | 3 | | **Anorexia** | MAS3 | 0.47 | 0.16 | 0.62 | 0.041 | * | 22 | v | 35 | |  | AQ+SP | 0.38 | 0.11 | 0.66 | 0.031 | * | 7 | v | 25 | |  | AQ+AS3 | 0.39 | 0.12 | 0.70 | 0.036 | * | 7 | v | 24 | |  | AL | 0.48 | 0.26 | 0.89 | 0.012 |  | 25 | v | 67 | | **Vomiting** | MAS3 | 0.30 | 0.15 | 0.58 | 0.000 | * | 30 | v | 14 | |  | AQ+SP | 0.31 | 0.14 | 0.70 | 0.003 | * | 8 | v | 26 | |  | AQ+AS3 | 0.39 | 0.16 | 0.91 | 0.025 | * | 8 | v | 19 | |  | AL | 0.85 | 0.45 | 1.60 | 0.609 |  | 21 | v | 24 | | **Sleeping** | MAS3 | 0.62 | 0.31 | 1.23 | 0.167 |  | 18 | v | 18 | |  | AL |  |  |  |  |  |  |  |  | | **Nightmare** | MAS3 | 0.47 | 0.41 | 5.36 | 0.535 |  | 1 | v | 2 | | **Dermatological** | MAS3 | 0.55 | 0.38 | 0.78 | 0.002 | * | 75 | v | 84 | |  |  |  |  |  |  |  |  |  |  | |  |  |  |  |  |  |  |  |  |  | | **ADULTS** |  |  |  |  |  |  |  |  |  | | **Diarrhoea** | MAS3 | 3.11 | 2.31 | 4.18 | 0.000 | * | 248 | v | 83 | | **Nausea** | MAS3 | 0.57 | 0.27 | 0.66 | 0.006 | * | 55 | v | 58 | | **Anorexia** | MAS3 | 1.22 | 0.80 | 1.87 | 0.356 |  | 78 | v | 50 | | **Vomiting** | MAS3 | 0.72 | 0.46 | 1.15 | 0.168 |  | 42 | v | 38 | | **Sleeping** | MAS3 | 0.51 | 0.27 | 0.66 | 0.003 | * | 49 | v | 45 | | **Nightmare** | MAS3 | 0.19 | 0.04 | 0.95 | 0.028 | * | 2 | v | 9 | | **Dizziness** | MAS3 | 0.44 | 0.27 | 0.70 | 0.000 | * | 52 | v | 63 | | **Dermato** | MAS3 | 0.60 | 0.46 | 0.79 | 0.000 | * | 156 | v | 157 | | **Palpitation** | MAS3 | 0.42 | 0.27 | 0.66 | 0.000 | * | 36 | v | 54 | | **Weakness** | MAS3 | 0.90 | 0.58 | 1.40 | 0.646 |  | 59 | v | 42 | | **Muscle pain** | MAS3 | 0.32 | 0.18 | 0.59 | 0.000 | * | 19 | v | 39 | | **Joint pain** | MAS3 | 0.94 | 0.53 | 1.66 | 0.835 |  | 32 | v | 25 | | **hearing** | MAS3 | 0.36 | 0.22 | 0.61 | 0.000 | * | 29 | v | 40 | | | --- | --- | --- | --- | --- | --- | --- | --- | --- | --- | --- | --- | --- | --- | --- | --- | --- | --- | --- | --- | --- | --- | --- | --- | --- | --- | --- | --- | --- | --- | --- | --- | --- | --- | --- | --- | --- | --- | --- | --- | --- | --- | --- | --- | --- | --- | --- | --- | --- | --- | --- | --- | --- | --- | --- | --- | --- | --- | --- | --- | --- | --- | --- | --- | --- | --- | --- | --- | --- | --- | --- | --- | --- | --- | --- | --- | --- | --- | --- | --- | --- | --- | --- | --- | --- | --- | --- | --- | --- | --- | --- | --- | --- | --- | --- | --- | --- | --- | --- | --- | --- | --- | --- | --- | --- | --- | --- | --- | --- | --- | --- | --- | --- | --- | --- | --- | --- | --- | --- | --- | --- | --- | --- | --- | --- | --- | --- | --- | --- | --- | --- | --- | --- | --- | --- | --- | --- | --- | --- | --- | --- | --- | --- | --- | --- | --- | --- | --- | --- | --- | --- | --- | --- | --- | --- | --- | --- | --- | --- | --- | --- | --- | --- | --- | --- | --- | --- | --- | --- | --- | --- | --- | --- | --- | --- | --- | --- | --- | --- | --- | --- | --- | --- | --- | --- | --- | --- | --- | --- | --- | --- | --- | --- | --- | --- | --- | --- | --- | --- | --- | --- | --- | --- | --- | --- | --- | --- | --- | --- | --- | --- | --- | --- | --- | --- | --- | --- | --- | --- | --- | --- | --- | --- | --- | --- | --- | --- | --- | --- | --- | --- | --- | --- | --- | --- | --- | --- | --- | --- | --- | --- | --- | --- | --- | --- | --- | --- | --- | --- | --- | --- | --- | --- | --- | --- | --- | --- | --- | --- | --- | --- | --- | --- | --- | --- | --- | --- | --- | --- | --- | --- | --- | --- | --- | --- | --- | --- | --- | --- | --- | --- | --- | --- | --- | --- | --- | --- | --- | --- | --- | --- | --- | --- | --- | --- | --- | --- | --- | --- | --- | --- | --- | --- | --- | --- | --- | --- | --- | --- | --- | --- | --- | --- | --- | --- | --- | --- | --- | --- | --- | --- | --- | --- | --- | --- | --- | --- | --- | --- | --- | --- | --- | --- | --- | --- | --- | --- | --- | --- | --- | --- | --- | --- | --- | --- | --- | --- | --- | --- | --- | --- | --- | --- | --- | --- | --- | --- | --- | --- | --- | --- | --- | --- | --- | --- | --- | --- | --- | --- | --- | --- | --- | --- | --- | --- | --- | --- | --- | --- | --- | --- | --- | --- | --- | --- | --- | --- | --- | --- | --- | --- | |  |
| --- | --- | --- | --- | --- | --- | --- | --- | --- | --- | --- | --- | --- | --- | --- | --- | --- | --- | --- | --- | --- | --- | --- | --- | --- | --- | --- | --- | --- | --- | --- | --- | --- | --- | --- | --- | --- | --- | --- | --- | --- | --- | --- | --- | --- | --- | --- | --- | --- | --- | --- | --- | --- | --- | --- | --- | --- | --- | --- | --- | --- | --- | --- | --- | --- | --- | --- | --- | --- | --- | --- | --- | --- | --- | --- | --- | --- | --- | --- | --- | --- | --- | --- | --- | --- | --- | --- | --- | --- | --- | --- | --- | --- | --- | --- | --- | --- | --- | --- | --- | --- | --- | --- | --- | --- | --- | --- | --- | --- | --- | --- | --- | --- | --- | --- | --- | --- | --- | --- | --- | --- | --- | --- | --- | --- | --- | --- | --- | --- | --- | --- | --- | --- | --- | --- | --- | --- | --- | --- | --- | --- | --- | --- | --- | --- | --- | --- | --- | --- | --- | --- | --- | --- | --- | --- | --- | --- | --- | --- | --- | --- | --- | --- | --- | --- | --- | --- | --- | --- | --- | --- | --- | --- | --- | --- | --- | --- | --- | --- | --- | --- | --- | --- | --- | --- | --- | --- | --- | --- | --- | --- | --- | --- | --- | --- | --- | --- | --- | --- | --- | --- | --- | --- | --- | --- | --- | --- | --- | --- | --- | --- | --- | --- | --- | --- | --- | --- | --- | --- | --- | --- | --- | --- | --- | --- | --- | --- | --- | --- | --- | --- | --- | --- | --- | --- | --- | --- | --- | --- | --- | --- | --- | --- | --- | --- | --- | --- | --- | --- | --- | --- | --- | --- | --- | --- | --- | --- | --- | --- | --- | --- | --- | --- | --- | --- | --- | --- | --- | --- | --- | --- | --- | --- | --- | --- | --- | --- | --- | --- | --- | --- | --- | --- | --- | --- | --- | --- | --- | --- | --- | --- | --- | --- | --- | --- | --- | --- | --- | --- | --- | --- | --- | --- | --- | --- | --- | --- | --- | --- | --- | --- | --- | --- | --- | --- | --- | --- | --- | --- | --- | --- | --- | --- | --- | --- | --- | --- | --- | --- | --- | --- | --- | --- | --- | --- | --- | --- | --- | --- | --- | --- | --- | --- | --- | --- | --- | --- | --- | --- | --- | --- | --- | --- | --- | --- | --- | --- | --- | --- | --- | --- | --- | --- | --- | --- | --- | --- | --- | --- | --- | --- | --- | --- | --- | --- | --- | --- | --- | --- | --- | --- | --- | --- | --- | --- | --- | --- | --- | --- | --- | --- | --- | --- |

Note: The risk of adverse event appearance after the start of dihydroartemisinin-piperaquine treatment in children (<15y) and adults who did not present this symptom on admission versus comparators in comparative studies. 95% confidence intervals (CI) are calculated for the odds ratio (OR)
